# Public health risk stratification using hybrid machine learning: a reproducible analysis of performance, stability, and risk attribution

**DOI:** 10.3389/fbinf.2026.1803572

**Published:** 2026-04-23

**Authors:** Alejandro Cabrera-Andrade, Ana Karina Zambrano, Joselin García-Ortiz, William Villegas-Ch

**Affiliations:** 1 Carrera de Enfermería, Facultad de Ciencias de la Salud, Universidad de las Américas, Quito, Ecuador; 2 Grupo de Bio-Quimioinformática, Universidad de las Américas, Quito, Ecuador; 3 Centro de Investigación Genética y Genómica, Facultad de Ciencias de la Salud Eugenio Espejo, Universidad UTE, Quito, Ecuador; 4 Escuela de Ingeniería en Ciberseguridad, Facultad de Ingeniería y Ciencias Aplicadas, Universidad de Las Américas, Quito, Ecuador

**Keywords:** artificial intelligence, explainable risk attribution, hybrid machine learning, public health data integration, risk stratification

## Abstract

Risk stratification in public health involves organizing heterogeneous health-related signals into consistent representations that support population-level analysis. In large-scale datasets, such as National Health and Nutrition Examination Survey (NHANES) and Behavioral Risk Factor Surveillance System (BRFSS), the integration of clinical, biometric, behavioral, and self-reported variables introduces structural variability that challenges conventional modeling approaches. This study proposes a hybrid learning framework that combines linear and nonlinear components to analyze induced risk representations derived from multidimensional health data. The model is evaluated using NHANES 2017–2018, BRFSS 2019, and an Integrated Public Health Dataset constructed through semantic harmonization of both sources. The experimental design is based on a controlled formulation in which a continuous risk index is constructed from the available variables and discretized into ordinal classes using quantiles, enabling systematic analysis of how models approximate structured partitions of the input space rather than predicting independent clinical outcomes. The results show that the hybrid scheme maintains consistent macro F1 and macro-ROC-AUC values across all scenarios with low fold-to-fold variability, reflecting the regularity of the induced class structure rather than predictive generalization. Attribution analysis reveals that the organization of the risk representation varies according to the nature of the data, with concentrated patterns in clinical signals, distributed contributions in behavioral variables, and intermediate structures in the integrated dataset. These findings demonstrate that hybrid schemes provide a stable and interpretable framework for analyzing the structural organization of risk in heterogeneous public health data.

## 1 Introduction

Risk stratification in public health involves assigning individuals or populations to ordered categories based on heterogeneous health-related signals. In large-scale observational datasets, such as National Health and Nutrition Examination Survey (NHANES) and Behavioral Risk Factor Surveillance System (BRFSS), these signals include biometric measurements, clinical conditions, behavioral factors, and demographic attributes, which differ in resolution, reliability, and semantic structure. The integration of these heterogeneous sources introduces significant methodological challenges, particularly when constructing consistent risk representations that remain stable across datasets with distinct statistical properties Paredes et al. (2024), Trujillano et al. (2025).

Traditional approaches to risk stratification have relied on deterministic rules or statistical models defined for specific clinical outcomes. More recent work has incorporated machine learning techniques to capture nonlinear interactions among risk factors. However, a substantial portion of the literature focuses on prediction tasks tied to predefined clinical labels, often within single datasets or controlled cohorts. This emphasis limits the understanding of how different types of signals contribute to the organization of risk when the objective is not outcome prediction, but the construction of consistent representations across heterogeneous data sources Khan et al. (2025), Shah et al. (2025).

Another limitation of existing studies is the frequent reliance on restricted-access datasets, which constrains reproducibility and hinders systematic evaluation under controlled conditions. In addition, the lack of standardized formulations for risk representation makes it difficult to compare results across studies, particularly when different definitions of “risk” are implicitly embedded in the modeling process. These limitations highlight the need for methodological frameworks that enable reproducible analysis of risk structures using open datasets and explicitly defined formulations Paredes et al. (2024), Zhang et al. (2025).

From an algorithmic perspective, risk stratification can be formulated as a representation problem, in which the objective is to approximate structured partitions of the input space rather than predict independent outcomes. In this formulation, the target variable is not externally defined. Still, it is constructed from the same underlying signals through a controlled transformation, enabling the analysis of induced risk structures under reproducible conditions. Within this perspective, hybrid learning schemes that combine linear and nonlinear components offer flexibility to capture both simple and complex relationships in heterogeneous data. However, empirical evidence remains limited on how such hybrid architectures perform when applied consistently across multiple public health datasets with varying signal characteristics Wang et al. (2025).

This study addresses this gap by proposing a hybrid risk stratification framework evaluated on NHANES 2017–2018 Yuan et al. (2024), BRFSS 2019 Budhathoki et al. (2023), and an Integrated Public Health Dataset constructed through semantic harmonization of both sources. The experimental design is based on a controlled formulation in which a continuous risk index is constructed from the available signals and discretized into ordinal classes using quantiles. This design enables systematic analysis of how learning-based models approximate induced risk structures while ensuring reproducibility through open datasets and clearly defined preprocessing and evaluation protocols.

The contribution of this work lies in the integrated evaluation of performance, stability, and attribution within a unified methodological framework. Rather than focusing on predictive accuracy, the study provides evidence on how heterogeneous signals organize risk representations across datasets and how hybrid models distribute influence within the induced structure. This perspective enables a consistent analysis of risk stratification as a structural and algorithmic problem, which is particularly relevant in exploratory public health scenarios where predefined outcome labels may be unavailable or may not capture the full complexity of the data.

## 2 Literature review

Health risk stratification has evolved from classical statistical models to machine learning architectures capable of processing heterogeneous variables and capturing nonlinear relationships. Despite this progress, recent literature reveals that methodological development has been primarily oriented toward predictive performance, with limited emphasis on how risk is structurally represented. Most studies frame risk stratification as a supervised learning task, where models are trained to predict predefined clinical outcomes, implicitly assuming that the target variable fully captures the notion of risk.

Traditional models, such as logistic regression, continue to serve as baseline approaches due to their interpretability and statistical stability, although their capacity to model complex interactions remains limited in high-dimensional settings Boakye et al. (2025), Trujillano et al. (2025). Notably, Boakye et al. (2025) demonstrate that, in certain domains, the performance gap between logistic regression and more complex machine learning models is marginal, raising questions about the practical benefits of increased model complexity when methodological gains are not clearly justified.

In contrast, a large body of work reports improved predictive performance through ensemble methods, gradient boosting, and hybrid architectures, particularly in cardiovascular risk and mortality prediction Chen et al. (2023), Shah et al. (2025). These approaches integrate clinical, biometric, and behavioral variables without imposing linear assumptions, enabling more flexible modeling of complex relationships. However, their evaluation is typically limited to aggregate performance metrics such as AUC or F1-score, without explicitly analyzing how individual variables contribute to risk construction or how these contributions behave under varying data conditions.

Hybrid models combining statistical, kernel-based, and neural approaches have emerged as a dominant paradigm in recent studies Khan et al. (2025), Nguyen et al. (2025), Shah et al. (2025). These models often achieve high predictive performance, sometimes approaching saturation levels Nguyen et al. (2025). Nevertheless, from a methodological perspective, these results are frequently presented without sufficient discussion of reproducibility, model stability, or sensitivity to data heterogeneity. As a result, improvements in predictive accuracy do not necessarily translate into a deeper understanding of the underlying risk structure.

A recurring methodological trend is the incorporation of optimization and feature selection techniques to enhance model performance and generalization. Huang et al. (2025) show that combining Bayesian optimization with explainable models can improve both accuracy and computational efficiency, while Wang et al. (2025) propose formal variable selection frameworks that control the false discovery rate. These contributions highlight the importance of methodological design beyond model selection, emphasizing the role of variable interactions and optimization strategies in shaping predictive outcomes. However, they remain embedded within supervised paradigms and do not explicitly address how risk is represented independently of prediction.

Explainability has also gained increasing attention, primarily through *post hoc* methods such as SHAP and LIME, which aim to identify dominant predictors and local decision patterns Shah et al. (2025), Talukder et al. (2025). While these approaches improve transparency, they are generally applied after model training and do not form part of the core modeling process. Consequently, explainability is often treated as a validation layer rather than as an intrinsic component of risk stratification, limiting its capacity to inform structural interpretations of risk or support robust decision-making in real-world settings.

Another important trend is the integration of multidimensional data sources, including clinical, biochemical, behavioral, and administrative variables Seyam (2025), Veledar et al. (2025). This multidimensionality enhances predictive capability but introduces additional challenges related to data heterogeneity, bias, and model generalization across populations. Although these limitations are acknowledged in the literature, they are typically addressed through standard techniques such as cross-validation or hyperparameter tuning, without formal mechanisms to evaluate the stability or consistency of risk representations across datasets.

Existing studies demonstrate that machine learning and hybrid approaches have been widely applied to health risk stratification. However, a critical methodological gap remains. Current approaches largely conflate risk representation with outcome prediction, relying on predefined labels as proxies for risk while neglecting the explicit modeling of latent risk structures. Furthermore, they rarely assess whether learned risk patterns are stable across heterogeneous datasets or whether interpretability mechanisms remain consistent under different modeling conditions. This gap highlights the need for frameworks that decouple the construction of risk representations from outcome prediction, enabling a structured analysis of how risk is formed, how it behaves under varying data distributions, and how it can be interpreted consistently across heterogeneous data sources.

Several recent studies have extensively employed NHANES and BRFSS datasets for predictive health risk modeling, reinforcing their relevance in data-driven public health research. For instance, NHANES has been widely used to develop machine learning models for hypertension, cardiovascular disease, and metabolic risk prediction, often integrating clinical, laboratory, and demographic variables to achieve high predictive performance Guo et al. (2023), Ahiduzzaman and Hasan (2025), Lu et al. (2025). These studies typically rely on supervised learning frameworks, where models are trained to predict predefined clinical outcomes using evaluation metrics such as accuracy, F1-score, and ROC-AUC.

Similarly, BRFSS-based studies have focused on large-scale population-level risk prediction, particularly for chronic conditions such as diabetes and cardiovascular disease, leveraging behavioral and self-reported variables Muhammad et al. (2025). These approaches emphasize predictive accuracy and model interpretability, frequently employing ensemble methods and explainable AI techniques to identify dominant risk factors. Despite their methodological diversity, both NHANES- and BRFSS-based studies predominantly frame risk stratification as an outcome prediction task, without explicitly modeling the structural properties of the underlying risk space or evaluating its internal consistency.

While NHANES and BRFSS are both widely used in public health research, prior studies have predominantly analyzed these datasets in isolation. NHANES-based approaches typically rely on high-resolution clinical and laboratory measurements. In contrast, BRFSS-based studies are centered on self-reported behavioral and health-perception variables, resulting in fundamentally different statistical and semantic structures. The direct integration of these datasets into a unified risk stratification framework remains limited, primarily due to challenges in semantic harmonization, differences in variable definitions, and inconsistencies in measurement granularity. These limitations motivate the formulation of the proposed framework, which explicitly addresses these challenges through a unified and controlled representation of heterogeneous public health data.

## 3 Materials and methods

### 3.1 Methodological design and data sources

This study adopts a computational research design to develop and evaluate a hybrid machine learning framework for health risk analysis across heterogeneous data settings. Rather than focusing exclusively on clinical outcome prediction, the proposed approach explicitly distinguishes between two complementary dimensions of risk analysis: (i) the construction of a latent, data-driven risk representation derived from multidimensional health variables, and (ii) the prediction of clinically relevant outcomes based on such representations. This separation is formally defined in the subsequent sections, ensuring that representation and prediction are treated as independent components within the methodological pipeline.

The methodological design is particularly well-suited to open-access population datasets, where variables exhibit substantial heterogeneity in statistical distributions, semantic interpretations, and measurement scales. In such settings, the primary objective is not direct clinical validation but the formalization of reproducible, robust analytical pipelines that integrate diverse sources of information. This perspective aligns with recent studies emphasizing methodological rigor, interpretability, and controlled modeling in complex health data environments Paredes et al. (2024), Zhang et al. (2025).

The justification for this design lies in the multidimensional nature of health risk, which involves interactions among clinical, biometric, and behavioral variables collected through heterogeneous protocols. This heterogeneity introduces non-homogeneous distributions, missing values, and nonlinear dependencies that limit the effectiveness of single-model approaches. Consequently, the proposed framework prioritizes the controlled construction of an analytical cohort and the explicit representation of risk as stages that precede supervised learning, ensuring that the data’s intrinsic structure drives the learning architecture.

**FIGURE 1:**
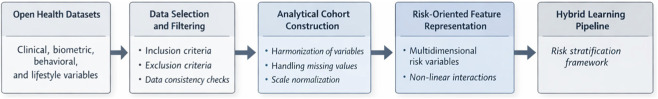
Overview of the methodological design and data integration process for dual-stage health risk analysis.

Figure 1 presents the methodological design and data integration process adopted in this study, illustrating the transition from open-access datasets to a structured analytical cohort and subsequently to a risk-oriented representation used as input for the hybrid learning framework.

The process begins with selecting publicly available health datasets from open repositories. These datasets include clinical, biometric, behavioral, and lifestyle variables, which are considered jointly to capture the multidimensional nature of health risk. The use of multiple data sources prioritizes representational diversity rather than increasing sample size.

A structured data selection and filtering stage is applied to ensure methodological consistency. Inclusion criteria retain records with valid and semantically interpretable values, while exclusion criteria remove duplicate entries, physiologically implausible observations, and records with excessive missingness. Consistency checks are performed to identify discrepancies in coding schemes and to ensure compatibility across variables originating from different sources.

The filtered data are used to construct the analytical cohort through a harmonization process that aligns equivalent variables and resolves structural heterogeneity. This process includes normalization of numerical variables to ensure scale comparability, systematic treatment of missing values, and reconciliation of attributes with equivalent semantic meaning across datasets. The resulting dataset is functionally coherent and suitable for methodological evaluation without assuming epidemiological representativeness.

To improve interpretability, the datasets used in this study include a combination of biometric, clinical, and behavioral variables explicitly defined in the feature engineering stage. In NHANES, the selected variables correspond to structured clinical and laboratory measurements such as glucose levels, blood pressure, and cholesterol indicators. In BRFSS, the variables are primarily derived from self-reported information, including behavioral patterns and indicators of chronic conditions such as smoking, physical activity, hypertension, and diabetes.

The distribution of the induced risk classes is defined by quantile-based discretization of the continuous risk index, resulting in three ordinal categories with balanced representation. Additionally, missing data handling is incorporated within the preprocessing pipeline through threshold-based exclusion and statistical imputation, ensuring consistency between data preparation and subsequent modeling stages without introducing additional assumptions beyond the observed data. A detailed statistical characterization of the variables is beyond the scope of this study, as the focus is on the structural analysis of induced risk representations rather than descriptive population analysis.

Once the analytical cohort is established, the variables are transformed into a risk-oriented representation that captures latent structural patterns without imposing predefined outcome labels. This transformation preserves multidimensional relationships and enables the modeling of complex interactions among variables. No supervised learning is applied at this stage, ensuring that the representation reflects the data’s intrinsic structure rather than being target-driven.

### 3.2 Feature engineering and risk variable modeling

Feature engineering is formulated as a risk-modeling stage in which variables are selected, transformed, and structured to represent multidimensional health-risk signals. The objective is not solely predictive optimization, but the construction of a consistent variable space that preserves clinical, behavioral, and population-level information while enabling interaction across heterogeneous attributes.

The variables are derived from two complementary open-access datasets: NHANES 2017–2018 and BRFSS 2019 Steele et al. (2023), Wesselbaum (2024). NHANES provides objective clinical and laboratory measurements, including age, sex, body mass index (BMI), systolic and diastolic blood pressure, glucose levels, total cholesterol, and related biochemical indicators. These variables characterize physiological and metabolic conditions with high measurement precision. BRFSS contributes large-scale self-reported data describing health conditions, behavioral patterns, and lifestyle factors, including chronic disease history, physical activity, tobacco use, and alcohol consumption.

The preprocessing pipeline was applied consistently across both datasets while accounting for their structural differences. In NHANES, continuous clinical and laboratory variables were filtered to remove physiologically implausible values and subsequently standardized using Equation 1. In BRFSS, categorical and ordinal variables derived from self-reported responses were harmonized into consistent numerical representations to ensure compatibility across survey coding schemes.

To enable integration between datasets, variables with equivalent semantic meaning were mapped into unified representations. Clinical conditions such as hypertension and diabetes, as well as behavioral indicators such as smoking and physical activity, were transformed into consistent binary or ordinal signals. This harmonization step ensured that the integrated dataset preserves semantic consistency while maintaining statistical comparability across sources.

Variable selection is performed by retaining attributes with direct or indirect relevance to population-level risk, prioritizing variables with consistent definitions across datasets and established use in epidemiological studies. Continuous variables $x_{j}$ , including biometric and laboratory measurements, are transformed through standardization as defined in Equation 1:

$$z_{j}=\frac{x_{j}-\mu_{j}}{\sigma_{j}}$$ (1)

where $\mu_{j}$ and $\sigma_{j}$ denote the empirical mean and standard deviation of variable $x_{j}$ . This transformation preserves relative variability and ensures scale invariance across heterogeneous attributes.

Categorical variables are encoded according to their semantic structure. Ordinal variables retain their inherent ordering, whereas binary indicators represent nominal variables without introducing artificial ordinal relationships. Binary health condition variables and behavioral indicators are preserved as discrete signals, maintaining their interpretability within the risk representation space Pierannunzi et al. (2013).

Missing data are treated as a structural component of the modeling process. For each variable, the proportion of missingness is computed, and variables exceeding a predefined threshold $\tau_{m}=0.3$ are excluded. For retained variables, missing values are imputed using distribution-consistent statistics, specifically the median for continuous variables and the mode for categorical variables, ensuring minimal distortion of the empirical distribution.

Each transformed variable is interpreted as a risk signal $s_{j}$ , defined as shown in Equation 2:

$$s_{j}=\phi_{j}\left(x_{j}\right),\;j=1,\ldots ,p$$ (2)

where $\phi_{j}\left(\cdot \right)$ denotes the transformation applied to variable $x_{j}$ , including normalization or categorical encoding. $\left\{s_{j}\right\}$ defines a multidimensional feature space in which each component corresponds to a normalized or encoded representation of an original attribute. This space is used as the input structure for risk formulation.

The application of the missingness threshold $\tau_{m}=0.3$ resulted in the exclusion of variables with substantial incompleteness, while preserving those with stable distributions across records. The retained variables exhibit low to moderate missingness, ensuring that the imputation process does not dominate the data’s statistical structure and that the resulting feature space remains representative of the original distributions.

### 3.3 Hybrid learning architecture and training strategy

The learning architecture is defined as a hybrid scheme operating on the risk-oriented feature space, integrating models with complementary representational properties. The input to the system corresponds to the transformed signal vector $x=\left(s_{1},\ldots ,s_{p}\right)$ , where each component represents a normalized or encoded risk signal.

The architecture comprises two classes of base models with distinct representational characteristics. The first class includes linear models that capture global relationships between variables and provide stable approximations under variations in data distribution. The second class includes nonlinear models with higher expressive capacity, enabling the identification of complex interactions among risk signals. In this implementation, the linear component is defined using logistic regression, while the nonlinear component is defined using a random forest model.

The selection of these models is motivated by their complementary inductive biases and interpretability within the proposed framework. Logistic regression provides a linear global approximation of the relationship between input signals and the induced risk structure, offering stability and transparency under variations in data distribution. In contrast, the random forest model captures nonlinear interactions and higher-order dependencies among variables without imposing parametric assumptions.

This combination enables the hybrid architecture to represent both structured linear trends and complex nonlinear patterns within a unified risk-oriented feature space. Rather than selecting models based solely on predictive performance, the choice is driven by the need to analyze how different representational assumptions influence the organization of the induced risk structure under controlled conditions.

**FIGURE 2:**
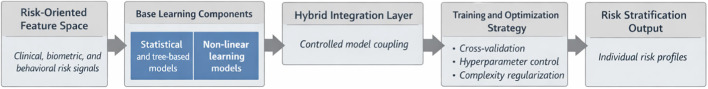
Hybrid learning architecture and training strategy for health risk modeling.

These complementary roles are illustrated in Figure 2, which presents the hybrid learning architecture and its training strategy. The integration of these components is formally defined by the weighted aggregation in Equation 4, which combines their outputs into a unified risk representation.

Let the output of each base model be defined as fi(x), as shown in Equation 3:

$$f_{i}\left(x\right),\;i=1,\ldots ,K$$ (3)

where $f_{i}\left(x\right)$ corresponds to the prediction of the i -th model applied to the input vector x . The hybrid integration is defined as a convex combination of these outputs, as expressed in Equation 4:

$$R\;\left(x\right)=\overset{K}{\underset{i=1}{\sum }}w_{i}f_{i}\left(x\right),\;\overset{K}{\underset{i=1}{\sum }}w_{i}=1,\;w_{i}\geq 0$$ (4)

where $w_{i}$ denotes the weight associated with the i -th model. The constraint $\sum_{i=1}^{K}w_{i}=1$ ensures that the aggregated output remains within a bounded representation space defined by the base models. This formulation preserves the relative contribution of each component while preventing dominance of a single model.

The weights $w_{i}$ are estimated via a constrained optimization procedure on the training set, minimizing a loss function defined over the target variable while preserving the normalization constraint. Each base model is trained independently using the same input representation, and their outputs are combined exclusively at the integration stage. This separation ensures that individual model learning and aggregation remain decoupled within the hybrid framework.

### 3.4 Risk score and outcome definition

The proposed framework distinguishes between two complementary components: a latent risk representation derived from multidimensional health variables and an outcome variable used for supervised evaluation. This distinction ensures that the risk representation is not constructed from the same target used in the predictive stage.

The latent risk representation corresponds to the aggregated output of the hybrid learning architecture defined in Equation 4. Let $x\in R^{d}$ denote the risk-oriented feature vector composed of transformed signals $s_{j}$ . The function R(x) is a continuous, data-driven risk score representing interactions among variables, biometric, and behavioral variables.

The supervised evaluation component is defined through an outcome variable y , constructed from clinically interpretable attributes present in the original datasets. Let y∈{0,1} denote a binary outcome derived from a physiological indicator using a threshold-based rule.

Formally, the outcome variable is defined as shown in Equation 5:

$$y=I\left(z_{k}>\tau \right),$$ (5)

where $z_{k}$ represents a selected clinical variable (e.g., glucose level or blood pressure), τ is a clinically relevant threshold, and I(⋅) denotes the indicator function. The variable $z_{k}$ is not modified during feature transformation and remains independent of the constructed risk representation. This definition ensures that the outcome variable is derived from original clinical measurements and is not constructed from the latent representation R(x) or from any transformation applied during feature engineering.

Although the induced risk representation and the input features originate from the same underlying variables, this formulation is explicitly treated as a controlled experimental design. The objective is not to predict independent clinical outcomes but to analyze the structural properties of a data-driven risk space. This distinction prevents unintended information leakage and ensures that representation learning and outcome evaluation remain formally separated within the methodological pipeline.

### 3.5 Training strategy, hyperparameter configuration, and complexity control

The training strategy is defined to ensure controlled optimization, reproducibility, and stability of the hybrid learning system when operating on heterogeneous health data. The procedure integrates model training, hyperparameter selection, and regularization within a unified framework. This organization ensures that parameter estimation and model evaluation remain formally separated throughout the learning process.

The dataset is partitioned into two disjoint subsets using stratified sampling, allocating 70% of the observations to the training set and 30% to the evaluation set. This partition preserves the distribution of risk levels across both subsets and ensures consistent evaluation on unseen data. The evaluation set is excluded from all training and hyperparameter tuning procedures to prevent information leakage during model selection.

Let $x\in R^{d}$ denote the risk-oriented feature vector and let $f_{i}\left(x\right)$ be the output of the i -th base model. Each function $f_{i}\left(x\right)$ represents the response of an individual learning component applied to the same input representation. The aggregated representation used during training corresponds to the hybrid formulation defined in Equation 4.

The optimization of the system is formulated as shown in Equation 6:

$$\min_{\Theta }\;L\left(R\left(x\right),y\right)+\lambda \Omega \left(\Theta \right)$$ (6)

where Θ denotes the set of model parameters and hyperparameters. The function L(⋅) represents the loss defined over the target variable, while Ω(Θ) corresponds to a regularization term. The parameter λ≥0 controls the trade-off between data fitting and model complexity.

Although the structural hyperparameters of the base models were fixed to ensure controlled comparison across datasets, preliminary experiments were conducted to evaluate different configurations. For the logistic regression model, variations in the regularization strength were explored over C∈{0.01,0.1,1,10} , confirming that higher iteration limits (max_iter≥3000) ensured stable convergence without affecting performance variability. For the random forest model, alternative configurations of n_estimators∈{200,500,800} and min_samples_leaf∈{1,3,5} were evaluated. The selected configuration ( n_estimators=500 , min_samples_leaf=3 ) provided a balance between model stability and computational efficiency across all datasets.

The final model configuration was selected based on cross-validated performance on the training set, using macro F1-score as the primary criterion. This selection strategy ensures that the reported results are not dependent on a single parameter setting but rather reflect a stable configuration validated across multiple data partitions within the proposed experimental pipeline.

Each base model is trained independently using the same input representation x . Their outputs are aggregated exclusively at the integration stage, ensuring separation between individual learning processes and the combination mechanism. Complexity control is implemented through the constrained weight search, fixed model capacity, and validation-based selection on the training subset.

Algorithm 1 summarizes the training procedure, including data partitioning, model fitting, output aggregation, and parameter selection.

Algorithm 1 **Hybrid training strategy with controlled complexity.**

1: **Input:** $D=\left\{\left(x_{i},y_{i}\right)\right\}$ ⊳ Risk-oriented dataset
2: $M=\left\{f_{1},f_{2},\ldots ,f_{K}\right\}$ ⊳ Base learning components
3: Λ ⊳ Regularization parameter
4: $H_{w}=\left\{0.0,0.1,\ldots ,1.0\right\}$ ⊳ Weight search space
5: r=0.7 ⊳ Training ratio
6: **Output:** R(x) ⊳ Risk stratification model
7: Split D into $D_{\text{train}}$ and $D_{\text{test}}$ using stratifiedsampling (70/30)
8: Initialize logistic regression with max_iter= 3000

9: Initialize random forest with n_estimators=500 and min_samples_leaf=3

10: **for all** weight configuration $w\in H_{w}$ **do**
11:  **for all** fold in five-fold cross-validation on $D_{\text{train}}$ **do**
12:   Train each base model $f_{i}$ on the training fold
13:   Compute partial outputs $f_{i}\left(x\right)$

14:   Aggregate outputs using Equation 4
15:   Evaluate validation loss: L= L(R(x),y)+Λ⋅Ω(Θ)

16:  **end for**
17:  Store average validation loss for configuration w

18: **end for**
19: Select $\Theta^{*}$ minimizing validation loss
20: Retrain base models and integration layer on full $D_{\text{train}}$ using $\Theta^{*}$

21: Evaluate R(x) on $D_{\text{test}}$

22: **return** trained risk stratification model R(x)

### 3.6 Explainability and risk attribution mechanisms

Explainability is implemented as a risk-attribution mechanism defined directly over the aggregated output of the hybrid model. Let $x\in R^{d}$ denote the risk-oriented feature vector and let R(x) be the continuous risk representation produced by the hybrid architecture. The attribution mechanism is defined on the trained function R(x) and evaluated over the transformed input signals.

The attribution is defined through an additive decomposition of the aggregated risk function, as shown in Equation 7:

$$R\left(x\right)=R_{0}+\overset{d}{\underset{j=1}{\sum }}\Phi_{j}$$ (7)

where $R_{0}$ denotes the baseline risk level and $\Phi_{j}$ represents the contribution associated with the j -th input signal. The contributions are computed over the transformed signals $s_{j}$ , ensuring consistency with the feature engineering stage. This decomposition preserves alignment between the attribution mechanism and the hybrid model output.

The term $\Phi_{j}$ is defined as the variation of the aggregated function R(x) under controlled perturbations of the corresponding signal. Let $x^{\left(j\right)}$ denote the vector obtained by replacing the j -th component with a reference value. The contribution is estimated as shown in Equation 8:

$$\Phi_{j}=R\left(x\right)-R\left(x^{\left(j\right)}\right)$$ (8)

The reference value is defined as the empirical median of each signal computed over the training dataset. This formulation captures the marginal effect of each signal on the aggregated risk function without altering the internal structure of the base models. The estimation is performed independently for each signal while preserving the original input structure.

The resulting attribution vector $\left\{\Phi_{j}\right\}$ defines a structured representation of signal contributions aligned with the hybrid model output. Each component is computed from the same aggregated function R(x) , ensuring consistency between architecture, training, and attribution. The attribution stage remains fully integrated within the methodological pipeline.

The attribution mechanism is defined as a deterministic transformation of the aggregated function R(x) , where each contribution is computed with respect to a fixed reference value. This formulation ensures that the resulting attribution is reproducible and consistent across instances, as it does not depend on stochastic sampling or external approximation procedures. By construction, the attribution process remains fully aligned with the hybrid model structure, enabling a direct interpretation of how individual signals contribute to the induced risk representation within a unified analytical framework.

The proposed attribution mechanism is structurally related to additive decomposition frameworks but does not rely on Shapley-value theory or on *post hoc* explanation methods such as SHAP or LIME. Instead, it is defined through a perturbation-based decomposition applied directly to the aggregated function R(x) , where each contribution $\Phi_{j}$ is computed as the variation in the model output under controlled modification of the corresponding input signal.

Unlike SHAP, which estimates feature contributions through marginal effects across coalitions of features and requires combinatorial approximations, the proposed approach operates without sampling over feature subsets or cooperative game-theoretic assumptions. This design ensures that the attribution remains fully consistent with the hybrid model’s structure and avoids introducing external approximation layers.

### 3.7 Experimental protocol and ethical considerations

The experimental protocol is designed to evaluate the risk-stratification capacity of the proposed hybrid framework under controlled, reproducible conditions. The design emphasizes methodological consistency across datasets and experimental configurations, ensuring that model behavior can be analyzed independently of specific data partitions. All experiments are conducted using exclusively publicly accessible data sources and a derived dataset constructed from them.

The experimental analysis is performed on two categories of datasets. The first category includes widely used public health datasets, specifically NHANES 2017–2018 and BRFSS 2019, which provide anonymized clinical, biometric, and behavioral variables. These datasets are selected due to their standardized collection protocols, population-scale coverage, and frequent use in health risk modeling studies. The second category corresponds to a derived dataset obtained by integrating, harmonizing, and transforming these sources. This dataset reorganizes existing variables without introducing additional observations or external information, enabling evaluation under a unified multidimensional representation while preserving data provenance.

The selection of a single-year subset for each dataset is motivated by the need to ensure temporal consistency and comparability across variables within a controlled experimental setting. Multi-year aggregation may introduce distributional shifts, changes in survey design, and inconsistencies in variable definitions, particularly in large-scale public health surveys. The chosen configuration, therefore, prioritizes structural coherence of the induced risk representation over temporal generalization, enabling a consistent evaluation of the proposed framework under fixed data conditions.

Data partitioning follows the strategy defined in the training procedure, ensuring consistency across all experimental scenarios. Stratified sampling is applied to preserve the distribution of risk levels, and the evaluation subset is strictly reserved for final performance assessment. This subset is not used during parameter tuning or model selection, preventing information leakage and ensuring unbiased evaluation.

The experimental process includes the complete execution of the methodological pipeline, including feature transformation, hybrid model training, and risk representation. Cross-validation is applied on the training subset for hyperparameter selection, ensuring that performance estimates remain independent of the evaluation data. In addition, multiple runs with consistent initialization are conducted to assess the stability of the learning process under controlled conditions.

The evaluation framework is defined according to the risk stratification objective. Standard performance metrics, including accuracy, precision, recall, and F1 score, are computed alongside the area under the receiver operating characteristic curve (ROC–AUC). Although these metrics are commonly used in supervised predictive settings, in this study, they are not intended to assess predictive performance over an independent clinical outcome.

Instead, they quantify the degree of consistency between the model outputs and the induced structure of the risk representation derived from the aggregated function R(x) . As the target variable is constructed through quantile-based discretization of the same underlying signals, these metrics reflect how accurately the models approximate the structural organization of the induced risk space under controlled experimental conditions.

From an ethical perspective, the study relies exclusively on anonymized and publicly available datasets. The data do not contain identifiable personal information, and no procedures involving human subjects are conducted. Consequently, the study does not require ethics committee approval or informed consent under the policies governing the use of public data.

## 4 Results

### 4.1 Dataset composition and experimental scenarios

Table 1 summarizes the final composition of the datasets used in the experimental scenarios, including the filtered dataset size, the number of variables per signal type, and the number of risk classes considered in each case.

**TABLE 1:** Dataset composition and experimental scenarios.

Dataset	Final records	Biometric variables	Clinical variables	Behavioral variables	Total variables	Risk classes
NHANES 2017–2018	9254	6	5	0	11	3
BRFSS 2019	418268	2	4	3	9	3
Integrated public health dataset	427522	2	4	3	9	3

After cleaning and variable selection, the NHANES 2017–2018 dataset retains 9,254 valid records and 11 variables. This scenario is characterized by continuous biometric measurements and binary clinical indicators derived from structured physiological assessments. Behavioral variables are not included due to the requirement of consistency across the selected signals.

The BRFSS 2019 dataset, with 418,268 records, represents a large-scale population scenario dominated by self-reported and behavioral variables. Its feature space comprises nine variables that combine clinical conditions, lifestyle factors, and ordinal proxies for physiological attributes.

The Integrated Public Health Dataset is constructed by harmonizing NHANES and BRFSS, yielding 427,522 unique records. The feature space is restricted to nine shared variables to ensure structural compatibility across sources. In all scenarios, risk stratification is derived from a continuous index constructed from the available variables and subsequently discretized into three ordinal classes using quantiles. These classes are not externally defined clinical categories but are induced from the empirical distribution of the data.

### 4.2 Analysis of induced risk representation

The evaluation metrics reported in this study, including Accuracy, F1-score, and ROC-AUC, are not interpreted as measures of predictive performance over an independent clinical outcome. Instead, they quantify the degree of consistency between the model outputs and the induced structure of the risk space defined by the aggregated function R(x) and its quantile-based discretization. Given that the target variable is derived from the same underlying signals, these metrics reflect how accurately the models capture the structural organization of the induced risk space under controlled experimental conditions, rather than their ability to generalize to external or unseen clinical scenarios. Accordingly, the reported values should be interpreted as indicators of internal structural coherence rather than predictive capability.

To analyze the behavior of the proposed framework beyond predictive performance, three complementary evaluations are conducted. These analyses assess the reconstructability of the induced risk classes, the alignment between the learned representation and the underlying risk score, and the model’s sensitivity to controlled perturbations of key variables.

Table 2 presents the comparison between a trivial baseline derived directly from the risk score and the learning-based models, allowing the evaluation of whether the induced classes can be reconstructed without learning. The trivial baseline achieves near-perfect performance across all datasets, reaching an accuracy of 1.000 in both BRFSS and the integrated dataset. This result is expected, as the target variable is constructed through quantile-based discretization of the same underlying risk score. Consequently, the baseline does not represent a predictive model but a direct reconstruction of the induced class boundaries, establishing an upper bound for this specific task. Since the target variable is derived from the same underlying signals through quantile discretization, high classification performance is expected and does not indicate predictive generalization. Instead, it reflects the deterministic relationship between the risk score and the induced class boundaries within this controlled formulation.

**TABLE 2:** Comparison between the trivial baseline and the learning-based models across datasets.

Dataset	Model	Accuracy	Precision	Recall	F1-score	ROC-AUC
NHANES 2017–2018	Trivial baseline	0.995	–	–	–	–
NHANES 2017–2018	Linear	0.821	0.632	0.393	0.399	0.791
NHANES 2017–2018	Non-linear	0.939	0.896	0.811	0.845	0.986
BRFSS 2019	Trivial baseline	1.000	–	–	–	–
BRFSS 2019	Linear	0.849	0.859	0.843	0.843	0.952
BRFSS 2019	Non-linear	0.969	0.968	0.968	0.968	0.998
Integrated public health dataset	Trivial baseline	1.000	–	–	–	–
Integrated public health dataset	Linear	0.990	0.990	0.990	0.990	0.998
Integrated public health dataset	Non-linear	0.978	0.978	0.978	0.978	0.999

For the trivial baseline, all evaluation metrics converge to identical values because the risk score is deterministic and determines the induced class labels. Therefore, only accuracy is reported for clarity when performance is perfect.

The learning-based models exhibit high but consistently lower performance than the baseline. In NHANES, the non-linear model achieves an accuracy of 0.939, indicating that it approximates the induced structure but does not fully replicate the exact quantile boundaries. In BRFSS and the integrated dataset, the performance gap is reduced, reflecting the increased regularity and scale of the data. These results indicate that the classification task is strongly governed by the induced structure of the risk score and should not be interpreted as an independent predictive task. This observation does not invalidate the proposed approach; rather, it redefines its scope: the objective is not to predict externally defined clinical outcomes but to model the internal structure of an induced risk space in a consistent and analyzable manner.

To determine whether the models replicate or transform this structure, Table 3 reports the correlation between the learned representation R(x) and the underlying risk score. The correlations are consistently high but remain below unity across all datasets. In NHANES, the non-linear model achieves a Pearson correlation of 0.88, significantly higher than the linear model (0.66), indicating that non-linear transformations capture additional structure beyond a direct mapping. In BRFSS and the integrated dataset, correlations stabilize around 0.85, suggesting convergence toward a common structural representation in large-scale scenarios.

**TABLE 3:** Correlation between the learned risk representation and the original risk score.

Dataset	Model	Pearson	Spearman
NHANES 2017–2018	Linear	0.66	0.72
NHANES 2017–2018	Non-linear	0.88	0.91
BRFSS 2019	Linear	0.85	0.86
BRFSS 2019	Non-linear	0.86	0.87
Integrated public health dataset	Linear	0.82	0.86
Integrated public health dataset	Non-linear	0.82	0.86

The absence of perfect correlation indicates that the learned representation does not collapse to the original risk score. In practical terms, this means that although the learned representation is strongly aligned with the original score, it cannot be reduced to a simple deterministic transformation of it. Instead, the models approximate the induced risk space through transformations influenced by feature interactions and normalization effects.

Table 4 presents the perturbation analysis, which evaluates the sensitivity of the learned representation to controlled changes in key variables. The results reveal distinct patterns across datasets. In BRFSS and the integrated dataset, perturbations produce consistent directional changes: increases in variables such as BMI and smoking, and reductions in physical activity, lead to higher predicted risk values. The proportion of positive changes exceeds 80% in most cases, indicating stable and monotonic behavior.

**TABLE 4:** Perturbation analysis of key variables and their impact on the learned risk representation.

Dataset	Variable	Abs. Mean ΔR	% positive	% negative
NHANES 2017–2018	Glucose	0.347	0.29	0.71
NHANES 2017–2018	BMI	0.036	0.53	0.47
NHANES 2017–2018	Systolic blood pressure (SBP)	0.092	0.61	0.39
BRFSS 2019	BMI	0.248	0.92	0.08
BRFSS 2019	Physical activity	0.131	0.98	0.02
BRFSS 2019	Smoking	0.088	0.95	0.05
Integrated public health dataset	BMI	0.229	0.83	0.17
Integrated public health dataset	Physical activity	0.172	0.85	0.15
Integrated public health dataset	Smoking	0.156	0.86	0.14
Integrated public health dataset	High blood pressure	0.285	0.60	0.40

Conversely, NHANES exhibits heterogeneous responses. Notably, perturbations in glucose levels result in inverse changes in the majority of instances, with more than 70% of cases showing a decrease in predicted risk. This behavior indicates that the model does not encode direct clinical causality but instead reflects the geometry of the transformed feature space and its interaction with the quantile-based discretization. The differences observed across datasets highlight the role of data scale and feature composition. Large-scale datasets such as BRFSS produce smoother and more stable risk surfaces, while smaller datasets such as NHANES exhibit local distortions and non-linear effects. The baseline, correlation, and perturbation analyses provide complementary evidence regarding the nature of the learned representation. While the baseline confirms the induced nature of the classification task, the correlation and perturbation analyses demonstrate that the model captures a transformed and internally consistent structure of the risk space.

This capability enables the systematic analysis of how variations in input variables influence the organization of the induced risk space, providing a framework for studying structural patterns that are not directly observable in the original risk score. These results demonstrate that the learned representation behaves as a structured mapping of the input space, with consistent sensitivity patterns under controlled perturbations.

### 4.3 Model behavior under induced risk stratification

The results reported in Table 5 describe the behavior of the linear and nonlinear models under an induced risk-stratification setting, where the target variable is obtained through quantile-based discretization of a continuous risk index. In this setting, the evaluation does not correspond to a conventional predictive task over externally defined outcomes, but to the extent to which the models approximate the induced class structure.

**TABLE 5:** Behavior of risk stratification models under induced class construction.

Dataset	Model	Accuracy	Precision (macro)	Recall (macro)	F1-score (macro)	ROC-AUC (macro)
NHANES 2017–2018	Linear	0.821	0.609	0.394	0.401	0.791
NHANES 2017–2018	Non-linear	0.947	0.895	0.841	0.864	0.987
BRFSS 2019	Linear	0.849	0.859	0.843	0.843	0.951
BRFSS 2019	Non-linear	0.987	0.986	0.986	0.986	0.999

In NHANES 2017–2018, the linear model achieves an accuracy of 0.821, but its macro recall decreases to 0.394 and its macro F1-score to 0.401, indicating limited balance across the induced classes. This behavior indicates that a linear decision surface is insufficient to reproduce the quantile-defined boundaries with comparable sensitivity across categories. The nonlinear model substantially improves all metrics, attaining an accuracy of 0.947, a macro precision of 0.895, a macro recall of 0.841, a macro F1-score of 0.864, and a ROC-AUC of 0.987. This pattern indicates that nonlinear transformations provide a closer approximation to the induced stratification in a dataset characterized by continuous physiological variables, binary clinical indicators, and lower sample density. Under this configuration, the hybrid scheme converges to the nonlinear solution, showing that the linear component does not contribute additional discriminative capacity in this scenario.

In BRFSS 2019, the linear model already exhibits high and balanced values across all metrics, with accuracy, macro precision, macro recall, and macro F1-score concentrated around 0.84–0.86 and a ROC-AUC of 0.951. This distribution indicates a more regular alignment between the feature space and the induced class organization. The nonlinear model intensifies this behavior, achieving an accuracy of 0.987 and macro precision, recall, and F1-score of 0.986, along with a ROC-AUC of 0.999. Under these conditions, the hybrid configuration is dominated by the nonlinear component, yielding behavior equivalent to the nonlinear estimator. The observed metrics correspond to the approximation of induced class boundaries derived from the risk score, rather than to predictive performance on externally defined outcomes.

**FIGURE 3:**
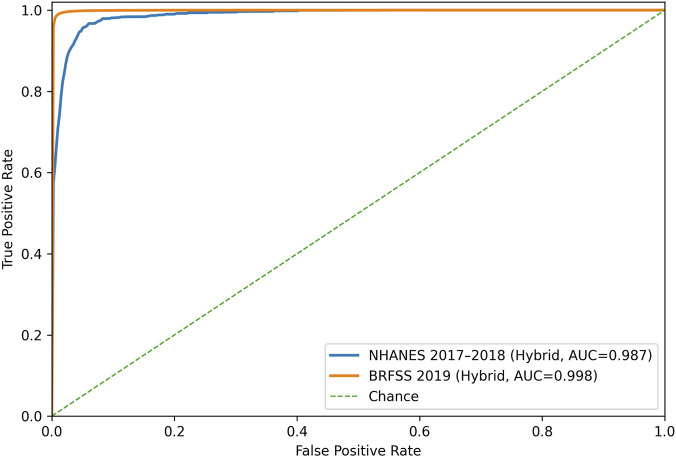
ROC curves of the hybrid risk stratification scheme evaluated independently on NHANES and BRFSS.

Figure 3 presents the macro-averaged ROC curves under a one-versus-the-rest scheme for the hybrid model, computed from the combined classification probabilities and evaluated independently in NHANES 2017–2018 and BRFSS 2019.

In both datasets, the curves rise sharply at low false-positive rates, indicating that the model assigns high probabilities consistent with the induced class ordering across a broad range of thresholds. This behavior reflects the hybrid configuration’s capacity to approximate the discretized class boundaries rather than to demonstrate independent predictive performance.

In NHANES, the ROC curve shows a steep initial rise followed by a more gradual approach to saturation. This shape indicates non-uniform separation between classes, which is coherent with the lower sample density and the greater heterogeneity introduced by continuous biometric measurements. In BRFSS, the curve remains close to saturation throughout most of the false-positive domain, indicating a more homogeneous and statistically stable structure in the induced risk space. This contrast between datasets is consistent with the larger scale of BRFSS and the aggregation of behavioral and clinical signals, which generate a smoother discrimination pattern under the induced stratification framework. The learned representation captures structural consistency in the discretized risk space without encoding independent predictive relationships over clinically defined outcomes.

### 4.4 Performance on the Integrated Public Health Dataset

Table 6 reports the behavior of the risk stratification scheme when applied to the Integrated Public Health Dataset using a 70/30 stratified partition. The evaluation includes macro-averaged metrics for the linear and nonlinear models, as well as the hybrid scheme, whose weights are determined exclusively on the training set via cross-validation.

**TABLE 6:** Performance on the derived integrated dataset.

Dataset	Model	Accuracy	Precision (macro)	Recall (macro)	F1-score (macro)	ROC-AUC (macro)
Integrated public health dataset	Linear	0.991	0.991	0.991	0.991	0.999
Integrated public health dataset	Non-linear	0.990	0.990	0.990	0.990	1.000
Integrated public health dataset	Hybrid (w = 0.4/0.6)	0.994	0.994	0.994	0.994	1.000

The linear model attains values close to 0.99 across all evaluation metrics, indicating that the induced class structure in the integrated feature space is highly regular. Under this configuration, a simple decision boundary is sufficient to approximate the quantile-based partitions of the risk score, reflecting reduced structural variability after harmonizing signals across datasets.

The nonlinear model exhibits virtually identical performance, with only marginal differences across metrics. This behavior indicates that the induced structure can be approximated without complex nonlinear transformations, suggesting that the integration process yields a smoother, more homogeneous representation of the risk space.

The hybrid scheme, whose weights are determined on the training set, selects a combination (0.4/0.6) between the linear and nonlinear components. The marginal improvement observed across metrics reflects the combination of probability estimates within a highly regular decision space, rather than the emergence of additional discriminative capacity. The observed values correspond to the approximation of induced class boundaries derived from the risk score rather than to predictive performance over externally defined outcomes.

**FIGURE 4:**
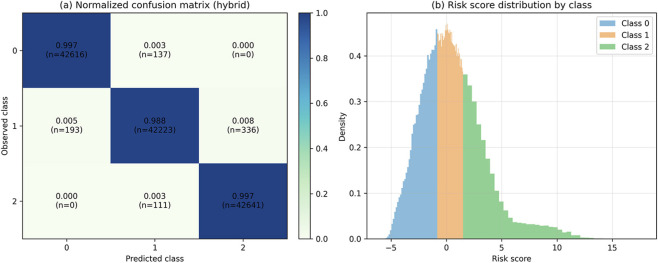
Evaluation of the hybrid risk stratification scheme on the Integrated Public Health Dataset: **(a)** normalized confusion matrix of the hybrid model evaluated on the test set; **(b)** risk_score distribution stratified by risk class.

Figure 4a presents the normalized confusion matrix of the hybrid model evaluated on the test set. The matrix exhibits a dominant diagonal across all three classes, with correct classification rates exceeding 0.98. Misclassifications are restricted to adjacent classes, primarily between classes 0 and 1 and between classes 1 and 2, with no direct confusion between the extreme categories. This pattern is consistent with the discretization of a continuous index into ordinal intervals, where classification uncertainty is concentrated in the transition regions between neighboring partitions.

Figure 4b shows the distribution of the risk score stratified by class. Negative values and moderate dispersion characterize the low-risk group, while the intermediate class is concentrated around the index’s central region, with controlled overlap with adjacent categories. The high-risk class is skewed toward positive values, exhibiting a longer right tail. These distributions define a continuous ordering of the risk index, and the overlap between classes corresponds to gradual transitions between contiguous segments rather than abrupt separations. The distributional structure of the risk score aligns with the observed classification patterns, reflecting the geometry of the induced risk space.

### 4.5 Stability and robustness analysis

The stability of the induced risk representation is evaluated through the variability of macro-averaged metrics across cross-validation folds, while maintaining the previously described experimental protocol. Table 7 reports the mean and standard deviation of Accuracy, macro F1-score, and macro ROC-AUC for each dataset and model configuration, allowing for an explicit assessment of the sensitivity of the induced structure to variations in data partitioning.

**TABLE 7:** Stability of performance metrics across cross-validation folds (TRAIN split).

Dataset	Model	Accuracy (mean ± std)	F1-score macro (mean ± std)	ROC-AUC macro (mean ± std)
NHANES 2017–2018	Linear	0.8169±0.0054	0.3898±0.0165	0.7856±0.0118
NHANES 2017–2018	Non-linear	0.9446±0.0049	0.8644±0.0143	0.9849±0.0045
BRFSS 2019	Linear	0.8475±0.0015	0.8418±0.0015	0.9502±0.0005
BRFSS 2019	Non-linear	0.9847±0.0010	0.9842±0.0010	0.9993±0.0001
Integrated public health dataset	Linear	0.9904±0.0005	0.9904±0.0005	0.9985±0.0002
Integrated public health dataset	Non-linear	0.9889±0.0009	0.9889±0.0009	0.9997±0.0000

In NHANES 2017–2018, the linear model yields a mean Accuracy of 0.8169 with a standard deviation of 0.0054, while the macro F1-score is 0.3898, with a higher relative variability of 0.0165. This pattern indicates that the induced class assignment is sensitive to variations in the training partitions, particularly in the presence of heterogeneous feature distributions and lower sample density. The nonlinear model reduces both the mean error and its variability, reaching an Accuracy of 0.9446 and a macro F1-score of 0.8644 with lower dispersion, indicating that nonlinear transformations capture a more stable representation of the induced structure under these conditions.

In BRFSS 2019, the models exhibit substantially reduced variability across folds. The linear model achieves a mean Accuracy of 0.8475 with a standard deviation of 0.0015, while the nonlinear model reaches 0.9847 with a standard deviation of 0.0010. The reduction in dispersion relative to NHANES reflects a more regular induced risk structure, in which variations in data partitioning produce only minor changes to the approximation of class boundaries.

The Integrated Public Health Dataset exhibits the lowest variability across all configurations. The linear model attains an Accuracy of 0.9904 with a standard deviation of 0.0005, while the nonlinear model reaches 0.9889 with a slightly higher deviation of 0.0009. These values indicate a highly consistent structural representation across folds, where the induced class organization remains stable regardless of the specific training subset.

**FIGURE 5:**
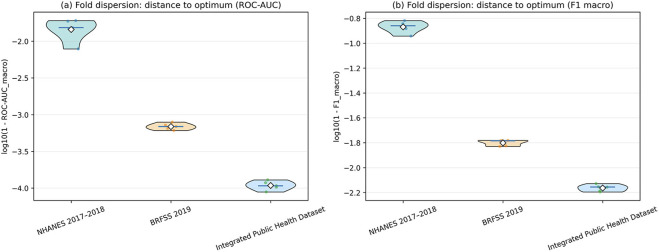
Stability analysis using cross-validation. **(a)** Distribution of the distance to the optimum expressed as $\log_{10}\left(1-{\text{ROC}-\text{AUC}}_{\text{macro}}\right)$ . **(b)** Distribution of the distance to the optimum for the macro F1-score, $\log_{10}\left(1-{\text{F}1}_{\text{macro}}\right)$ .

Figure 5 provides a complementary view of this behavior by representing the fold-to-fold variability as the logarithmic distance to the optimum. In Figure 5a, based on $\log_{10}\left(1-{\text{ROC}-\text{AUC}}_{\text{macro}}\right)$ , NHANES exhibits values centered around −1.9 with visible dispersion, indicating sensitivity in the separation between induced classes. In contrast, BRFSS concentrates around −3.2 with reduced spread, while the integrated dataset approaches values near −4.0 , forming a compact distribution that reflects minimal variation in the approximation of the induced boundaries.

Figure 5b, based on $\log_{10}\left(1-{\text{F}1}_{\text{macro}}\right)$ , shows a consistent pattern. NHANES presents values around −0.9 , with greater dispersion, indicating variability in the assignment across the induced classes. BRFSS shifts toward values near −1.8 with reduced variability, while the integrated dataset reaches values near −2.2 with minimal dispersion. The consistency between the two metrics indicates that the observed stability is not limited to class separability but also extends to the assignment within the induced class structure.

### 4.6 Risk attribution and explainability results

The functional decomposition of the hybrid model provides a representation of how the induced risk profile is distributed across clinical, biometric, and behavioral signals. Table 8 reports the normalized contributions of the most relevant signals for each dataset, reflecting the internal organization of the model rather than direct causal relationships.

**TABLE 8:** Top-20 risk attribution signals across datasets.

Signal	BRFSS 2019	Integrated public health dataset	NHANES 2017–2018	Mean across datasets
LBXGLU	—	—	0.9501	0.3167
physical_activity_signal	—	0.4219	—	0.1406
PHYSHLTH	0.3550	—	—	0.1183
MENTHLTH	0.3250	—	—	0.1083
smoking_signal	—	0.3070	—	0.1023
POORHLTH	0.2271	—	—	0.0757
highbp_bin	—	0.1788	—	0.0596
diabetes_bin	—	0.0531	—	0.0177
mi_bin	—	0.0199	—	0.0066
chd_bin	—	0.0192	—	0.0064
MCQ230A	—	—	0.0171	0.0057
RIAGENDR	—	—	0.0151	0.0050
_RACE	0.0141	—	—	0.0047
BPHIGH4	0.0104	—	—	0.0035
CHOLCHK2	0.0096	—	—	0.0032
_RACEGR3	0.0094	—	—	0.0031
_TOTINDA	0.0083	—	—	0.0028
MCQ160A	—	—	0.0069	0.0023
CHECKUP1	0.0054	—	—	0.0018
SMOKE100	0.0054	—	—	0.0018

In NHANES 2017–2018, the attribution structure is highly concentrated, with the LBXGLU variable accounting for the majority of the normalized contribution. This concentration reflects that the induced risk representation in this dataset is primarily organized around a reduced subset of signals, rather than indicating a direct causal dependence. Other variables, including MCQ230A, RIAGENDR, and MCQ160A, exhibit minimal contributions, indicating a steep hierarchy within the internal attribution structure.

In contrast, BRFSS 2019 presents a more distributed attribution pattern. Contributions are spread across multiple self-reported variables, including PHYSHLTH, MENTHLTH, and POORHLTH, each with comparable magnitudes. This distribution reflects a broader and more homogeneous organization of the induced risk representation, consistent with the nature of behavioral and survey-based signals.

The Integrated Public Health Dataset exhibits an intermediate structure characterized by composite and derived variables. Signals such as physical_activity_signal and smoking_signal concentrate a substantial proportion of the contribution, while binary indicators of chronic conditions contribute at lower but non-negligible levels. This pattern reflects the effect of signal harmonization, where multiple sources are aggregated into higher-level constructs that reorganize the attribution structure.

**FIGURE 6:**
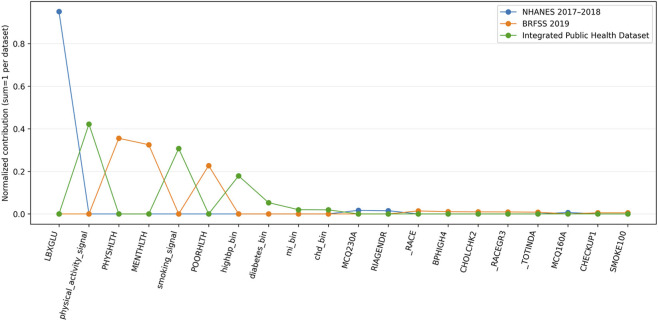
Relative organization of attribution signals across datasets.

Figure 6 illustrates these patterns by representing the normalized contributions across datasets. NHANES displays a sharply peaked profile dominated by a single signal, while BRFSS shows a smoother distribution with several signals contributing at similar levels. The integrated dataset shows intermediate peaks associated with composite variables, followed by a gradual decline in the magnitude of their contributions. Since each distribution is normalized independently, the figure reflects the internal organization of the attribution space within each dataset rather than direct comparisons of absolute importance.

Sorting by the “Mean Across Datasets” column highlights signals that appear across multiple sources, albeit with different relative contributions. Variables related to physical activity, smoking, and chronic conditions exhibit non-zero contributions across multiple datasets, whereas laboratory- or perception-based variables tend to dominate within a single source. This behavior indicates that the attribution structure is not fixed but depends on the composition and granularity of the available signals. The reported contributions correspond to an aggregated functional decomposition over the evaluation set and describe how the model distributes influence within the induced risk space, without implying causal relationships between individual variables and health outcomes.

## 5 Discussion

The results obtained in this study should be interpreted within the framework of an induced-risk representation rather than as evidence of predictive performance on independent clinical outcomes. The experimental design explicitly constructs the target variable from a continuous risk index derived from the same underlying signals used as input features. This formulation enables controlled analysis of how learning-based models approximate structured risk spaces, while avoiding unintended information leakage through strict separation of training and evaluation stages.

It is important to clarify that the term “continuous risk” in this study refers to the continuous nature of the constructed risk index derived from multidimensional input variables, and not to temporal or longitudinal modeling. The proposed framework operates on cross-sectional data, where each instance represents a static observation rather than a time-evolving profile. Therefore, the risk estimates do not correspond to predictions over future time horizons but rather to the structural organization of risk in the observed data at a given point.

Importantly, no data leakage is introduced in this process. The risk score used to define the induced classes is computed before data partitioning and remains fixed throughout the experimental pipeline. Model training and evaluation are performed on disjoint subsets, and the target variable is not recalculated or adapted based on model outputs. Furthermore, the cross-validation procedure is applied exclusively within the training set, ensuring that no information from the evaluation subset is used during model selection. Therefore, the observed high performance values result from the deterministic relationship between the constructed risk index and its discretized classes within a controlled formulation, rather than from unintended information leakage.

The evaluation does not aim to benchmark against state-of-the-art predictive models. Instead, it focuses on analyzing how representative model classes with distinct inductive biases approximate induced risk structures under a controlled formulation. In this setting, performance metrics reflect the deterministic organization of the constructed risk space rather than the comparative predictive capacity of different algorithms.

In this setting, high values of Accuracy, F1-score, and ROC-AUC are expected due to the deterministic relationship between the risk score and its discretized classes. Consequently, these metrics do not reflect predictive generalization but rather the degree to which the models reproduce the geometry of the induced class boundaries. This interpretation is supported by the baseline analysis, which demonstrates that a trivial mapping of the risk score achieves near-perfect classification, and by the correlation and perturbation analyses, which confirm that the learned representations approximate but do not trivially replicate the underlying risk structure.

The observed differences across datasets reflect variations in the statistical organization of the input space rather than differences in model capability. In NHANES, the concentration of attribution in a reduced subset of clinical variables indicates a highly structured and low-noise feature space. In contrast, the BRFSS dataset exhibits a more distributed attribution pattern, consistent with the variability inherent in self-reported behavioral signals. The integrated dataset represents an intermediate configuration, in which signal harmonization yields a more regular representation while preserving contributions from multiple domains. These patterns align with previous findings that highlight the distinct statistical properties of clinical and survey-based health data Steele et al. (2023), Pierannunzi et al. (2013).

From a methodological perspective, the proposed framework integrates hybrid learning, stability analysis, and functional attribution within a unified pipeline. Rather than improving predictive performance, this integration enables the characterization of how different types of signals organize the induced risk space under controlled conditions. The hybrid architecture provides flexibility in representing both linear and nonlinear relationships, while the stability analysis demonstrates that the induced structure remains consistent across data partitions. The attribution mechanism, defined by perturbing the aggregated function, provides a coherent interpretation of how the model distributes influence across signals without relying on external explanation methods such as SHAP or LIME.

The contribution of this work lies in formalizing a representation-driven approach to risk stratification, focusing on structural properties of the data rather than on outcome prediction. This perspective is particularly relevant in exploratory public health analysis, where heterogeneous data sources must be integrated and interpreted without assuming predefined causal relationships. By treating explainability as an intrinsic component of the modeling process, the proposed framework provides a consistent basis for analyzing how different signal types contribute to the organization of risk.

Several limitations must be considered. First, the risk variable’s induced nature implies that the results cannot be directly interpreted as predictive of real-world clinical outcomes. Second, the attribution values are normalized within each dataset, preventing direct comparison of magnitudes across sources. Third, the integration process assumes semantic compatibility between variables, which may introduce bias if definitions differ across datasets. Fourth, the analysis is restricted to static partitions and does not capture temporal dynamics, limiting its applicability in longitudinal settings. Finally, the study does not include validation on an external or independent dataset, which constitutes a methodological limitation. Although the experimental design is based on controlled, reproducible conditions using open-access data, the absence of external validation limits the generalizability of the findings beyond the specific induced formulation and data sources considered.

These considerations limit the scope of the proposed framework, which is better suited to structural analysis and exploratory modeling than to direct clinical decision-making. Future work should extend this approach to externally defined outcomes, incorporate longitudinal data, and evaluate robustness under alternative data integration strategies.

## 6 Conclusion

This work presents a representation-driven approach to risk stratification in public health, based on a hybrid learning scheme that combines linear and nonlinear components within a controlled experimental design. The proposed framework enables analysis of how learning-based models approximate structured risk spaces derived from heterogeneous health data, emphasizing the structural properties of the induced representation rather than predictive performance on externally defined outcomes.

The results show that the organization of the induced risk space varies according to the statistical nature of the underlying data. In NHANES 2017–2018, the attribution structure is highly concentrated, reflecting a feature space dominated by continuous clinical and biometric signals. In BRFSS 2019, the attribution becomes more dispersed, consistent with the inherent variability of self-reported behavioral data. The integrated dataset exhibits an intermediate configuration, in which semantic harmonization yields a more regular representation and shifts attribution toward composite signals derived from multiple sources.

From a methodological perspective, the framework integrates hybrid modeling, stability analysis, and functional attribution within a unified pipeline. This integration enables consistent characterization of how different signal types contribute to the induced risk structure under controlled conditions. The perturbation-based attribution mechanism provides a coherent interpretation of the model’s internal organization without relying on external explanation techniques or implying causal relationships between variables and health outcomes.

The findings highlight that high performance values observed in the experimental setting are a direct consequence of the induced formulation of the target variable. They should therefore be interpreted as an approximation of structured class boundaries rather than as evidence of predictive generalization. Within this context, the proposed approach is particularly suited for exploratory analysis of heterogeneous public health data, where the objective is to understand the organization of risk representations rather than to support individual clinical decision-making.

Future research should extend this framework to scenarios involving externally defined outcomes and longitudinal data, enabling the evaluation of temporal stability and generalization under real-world conditions. Additional work is also required to assess the robustness of the integration process under alternative harmonization strategies and to explore its applicability in distributed environments where data sharing is constrained.

## Data Availability

The datasets used and analyzed during the current study are publicly available. Specifically, the NHANES 2017–2018 dataset is available from the Centers for Disease Control and Prevention (https://wwwn.cdc.gov/nchs/nhanes/), and the BRFSS 2019 dataset is available from the Centers for Disease Control and Prevention (https://www.cdc.gov/brfss). Harmonizing variables from these sources constructed the integrated dataset used in this study. Any additional processed data or code supporting the findings of this study are available from the corresponding author upon reasonable request.
